# Why Is Tantalum Less Susceptible to Bacterial Infection?

**DOI:** 10.3390/jfb13040264

**Published:** 2022-11-22

**Authors:** Xin Chen, Yikang Bi, Moran Huang, Huiliang Cao, Hui Qin

**Affiliations:** 1 Department of Orthopedic Surgery, Shanghai Sixth People’s Hospital Affiliated to Shanghai Jiao Tong University School of Medicine, Shanghai 200233, China; 2 Department of Laboratory Medicine, The Affiliated Hospital of Xuzhou Medical University, Xuzhou 221002, China; 3 Department of Orthopedics, The Eighth People’s Hospital, Jiang Su University, Shanghai 200235, China; 4 Department of Orthopedics, Xuhui Branch of Shanghai Sixth People’s Hospital Affiliated to Shanghai Jiao Tong University School of Medicine, Shanghai 200235, China; 5 Department of Orthopedic Surgery, Shanghai General Hospital, Shanghai Jiao Tong University School of Medicine, Shanghai 201620, China; 6 Interfacial Electrochemistry and Biomaterials, Lab of Low-Dimensional Materials Chemistry, Key Laboratory for Ultrafine Materials of Ministry of Education, Shanghai Engineering Research Center of Hierarchical Nanomaterials, School of Materials Science and Engineering, East China University of Science & Technology, Shanghai 200237, China

**Keywords:** biomaterials, tantalum, race for the surface, implant-associated infection

## Abstract

Periprosthetic infection is one of the trickiest clinical problems, which often leads to disastrous consequences. The emergence of tantalum and its derivatives provides novel ideas and effective methods to solve this problem and has attracted great attention. However, tantalum was reported to have different anti-infective effects in vivo and in vitro, and the inherent antibacterial capability of tantalum is still controversial, which may restrict its development as an antibacterial material to some extent. In this study, the polished tantalum was selected as the experimental object, the implant-related tibia osteomyelitis model was first established to observe whether it has an anti-infective effect in vivo compared to titanium, and the early studies found that the tantalum had a lower infectious state in the implant-related tibia osteomyelitis model in vivo than titanium. However, further in vitro studies found that the polished tantalum was not superior to the titanium against bacterial adhesion and antibacterial efficacy. In addition, we focus on the state of interaction between cells, bacteria and materials to restore the internal environment as realistically as possible. We found that the adhesion of fibroblasts to tantalum was faster and better than that of titanium. Moreover, what is more, interesting is that, in the early period, bacteria were more likely to adhere to cells that had already attached to the surface of tantalum than to the bare surface of it, and over time, the cells eventually fell off the biomaterials and took away more bacteria in tantalum, making it possible for tantalum to reduce the probability of infection in the body through this mechanism. Moreover, these results also explained the phenomenon of the “race for the surface” from a completely different perspective. This study provides a new idea for further exploring the relationship between bacteria and host tissue cells on the implant surface and a meaningful clue for optimizing the preparation of antibacterial implants in the future.

## 1. Introduction

As an indispensable treatment for the restoration of function in modern medicine, biomaterial implants often fail because of implant-associated infection (IAI) [1]. Orthopedic IAI is a serious complication occurring in 1–2% after closed and in up to 30% after open fractures [2]. There are about 55,000 deaths from IAI annually in the USA, and the average cost of treating plant infections is up to $100,000 [1,3], which brings great pain and financial burden on patients. In addition, owing to the increase in antibiotic resistance and the difficulties in developing new antibiotics, hopeful strategies are urgently needed to change this situation [4,5]. In recent years, the antibacterial materials based on inorganic metal nanoparticles and their derivatives have been widely studied to prevent and treat biomaterial-related infections [6,7,8,9]. However, due to the inherent characteristics of most antibacterial nanomaterials, they not only have toxic effects on bacteria but also negatively affect the functions of normal human cells and tissues, thus limiting the application and clinical transformation of these new materials as implant coatings [10,11,12,13,14].

In recent years, clinically, titanium (Ti) and its derivatives have been widely used as implant materials in orthopedic surgery. Until now, the development of new titanium alloys has greatly optimized the mechanical properties of implants [15,16,17,18,19].Therefore, the focus of the urgent solution should be to select potential biomaterials to improve the effective antibacterial activity of implants and minimize their biological toxicity [20,21,22,23]. Tantalum (Ta) is considered a bioinert metal, which has attracted much attention because of its excellent chemical stability, biocompatibility, osteogenic activity, and corrosion resistance, and it has been widely applied in orthopedics and dentistry [24,25,26,27,28]. Moreover, Ta can effectively improve the capability of adhesion, proliferation, and differentiation of bone marrow mesenchymal stem cells and osteoblasts and promote osteogenic-related gene expression [29,30]. Especially porous Ta, as one of the excellent tantalum derivatives, has achieved gratifying results in clinical studies because of its appropriate elastic modulus and superior ability to induce bone regeneration [31,32,33]. In addition, tantalum and its derivatives are also reported to have effective antibacterial properties, but the inherent antibacterial activity of tantalum is still controversial. Different forms of tantalum, including metal ions and solid state, have been reported to have significant antibacterial activity [34,35]. Schildhauer et al. described that *S. aureus* had lower adhesion to pure tantalum than other common metal implant materials in vitro bacterial adhesion test [35]. At the same time, the structure of porous tantalum materials creates a suitable environment for bacterial colonization [36]. Moreover, in the in vitro experiments, porous tantalum did not show significant antibacterial properties [37]. However, it was demonstrated with satisfactory bony integration even if long-standing infection at the implantation site in the in vivo experiments [38]. Therefore, it is essential to further explore the inherent antibacterial properties of tantalum [26].

In this study, we first carried out in vivo tests and observed that the infection degree of the tantalum group was lighter than that of the titanium group, which prompted the authors to further continue in vitro tests to explore the possible mechanism behind these results. However, some interesting phenomena have been found in the author’s experiments; compared with titanium, fibroblasts not only adhered to tantalum more effectively. In the early period, bacteria also tend to adhere to the surface of cells rather than tantalum in the co-culture environment of cells and bacteria. The experimental results provide an idea for a further understanding of the relationship between host tissue and bacteria in the internal implant environment and can also be said to further explore the theory of “the race for the surface” from another point of view.

## 2. Materials and Methods

### 2.1. Sample Preparation and Characterization

Commercial pure titanium (Grade IV, ASTM) and tantalum (Ta2, YST 751-2011) were supplied by BAOTI Group (Baoji, Shaanxi, China)., China. In the in vitro tests, pure titanium and pure tantalum were prepared in 10 mm × 10 mm × 1 mm size and carefully polished to a mirror finish with abrasive paper and ultrasonically washed using ethanol and ultrapure water. The surface topography of Ta and Ti was evaluated using a field-emission scanning electron microscope (FE-SEM; S-4800, Hitachi, Japan). The depth profiles and chemical state of elements were examined by X-ray photoelectron spectroscopy. (XPS; PHI 5802, Physical Electronics Inc., Eden Prairie, MN, USA). The roughness of the surface in Ta and Ti has been evaluated by atomic force microscopy (AFM) before, as described in our previous paper [39]. Moreover, the contact-angle instrument (SL200B; Shanghai Solon Information Technology Co., Ltd., Shanghai, China) was used to measure the water contact angles of each sample. In addition, in the in vivo tests, the same materials were used to fabricate polished Kirschner wires with a length of 10 mm and a diameter of 1.2 mm.

### 2.2. Bacteria Preparation and Characterization

Freeze-dried *Staphylococcus aureus* (*S. a*; ATCC 43300) were get from the American Type Culture Collection (Manassas, VA, USA). Referring to the methodology of the author’s previous research [40], the Bacterial concentration used in the in vitro tests was 1 × 10^6^ colony forming units (CFUs)/mL in Trypticase Soy Broth (TSB; BD Biosciences, Franklin Lakes, NJ, USA), which was 1 × 10^5^ CFUs/mL in PBS in the in vivo tests. 

### 2.3. Implant-Related Tibia Osteomyelitis Model in Rats

The experimental protocol was approved by the Animal Care and Experiment Committee of Shanghai Sixth People’s Hospital, Affiliated with Shanghai Jiao Tong University School of Medicine (No: DWLL2018-0339). A total of 35 male Sprague Dawley rats (8 weeks) with an average weight of 215 g (180–255 g) were divided into three groups (Table 1). 0.6% pentobarbital sodium (0.9 mL/100 g body wt) was injected intraperitoneally into all the rats. The rats were treated as follows (Table 1). After shaving and disinfecting, a 0.5 cm long longitudinal incision was made on the tibial anteromedial side of the hind leg. A hole in the cancellous bone of the proximal metaphysis was drilled between the tibial tuberosity and the tibial plateau with a 1.0 mm-diameter Kirschner wire to enter the medullary cavity while ensuring the integrity of the surrounding periosteum. Then, 10 μL PBS or PBS containing *S. aureus* with a concentration of 1 × 10^5^ CFUs/mL was injected into the medullary cavity. According to the treatment, three groups of rats were divided into six groups according to their left and right legs. After bacterial inoculation, a polished metal Ta or Ti Kirschner wire (length:10 mm, diameter: 0.8 mm) was inserted into the medullary cavity. The subcutaneous tissue was irrigated carefully with a povidone-iodine solution, and the fascia and skin were closed in layers. After the operation, the animals were returned to different cages and allowed to move freely. The animals were allowed to bear weight and monitored daily. Buprenorphine was used as an analgesic for 2 days, but no antibiotics were used. 

#### 2.3.1. Radiographic Evaluation

Two weeks after the operation, the animals were photographed with high-resolution lateral X-ray films under general anesthesia using chloral hydrate. Three independent observers unaware of the study group evaluated three regions of interest (ROI): proximal epi-/metaphyseal area, diaphyseal region, and distal epi-/metaphyseal area. Radiographic assessment was based on the system used by Lucke et al. [41].

#### 2.3.2. Imaging Assay

At 14 days post-surgery, each rat (n = 5) was injected with 18.13–18.87 MBq (490–510 μCi) of 18F-FDG (Atomic Firm Sinovac Pharmaceutical co., LTD., Beijing, China) in 1.0 mL of saline via tail vein. The animals fasted for at least 8 h before the tracer injection. PET/CT scanning was performed at 40 min post tracer injection with GE Discovery VCT (General Electric Medical Systems, Milwaukee, WI, USA). The scanning conditions were as follows: CT scan, 120 kV and 80 mA, 64 slices, thickness 3.75 mm. PET scans were obtained in 3D, with 2.5 min/bed scanning time, and the animals were anesthetized by weight-adopted intraperitoneal injection of 0.6% pentobarbital sodium (0.9 mL/100 g body wt). Ordered subset expectation maximization (OSEM) was used to reconstruct the image iteratively. Attenuation correction was used in CT. 18F-FDG uptake was reported as the maximum standardized uptake value (SUVmax), which was calculated as the radioactivity of the region of interest (ROI) divided SUVmax for Ti+ *S. a* group, Ta+ *S. a* group were acquired, then the SUVmax differences between groups were compared.

#### 2.3.3. Microbiological Evaluation

After 14 days, 5 rats from III were sacrificed, and bilateral tibia was retrieved aseptically, followed by soft tissue removal and Kirschner wire explanted. To quantify bacteria adhesion, the explanted Kirschner wires were sonicated and vortexed to remove adhered bacteria in 4 mL PBS. The spread plate method was used to count the adhered bacteria. Then tibiae were selected randomly, frozen quickly, and ground into powder under aseptic conditions [42]. One tibial powder was agitated for 3 min in 2 mL PBS. After 10,000× *g* centrifugation for 15 s, the supernatant was continuously diluted (10 times), and the CFU/tibia was analyzed by the spread plate method.

#### 2.3.4. Histopathologic Evaluation

Masson’s trichrome staining was used to evaluate the morphological change on the tibia, and Giemsa staining was chosen to identify the residual bacteria. Five rats from Ⅲ were sacrificed, and Kirschner wires were explanted. The proximal tibia obtained from each group was decalcified in EDTA for 2 weeks, followed by dehydration and embedded in paraffin. Each specimen was cut to a 5 cm sagittal section and dyed by Masson’s trichrome staining and Giemsa staining, respectively.

### 2.4. In Vitro Antibacterial Assay

A total of 1 mL of prepared *S. aureus* suspension (1 × 10^6^ CFU/mL) was added into the 24-well plate containing Ti and Ta plates and cultured for different periods (3, 6, 12, and 24 h) at 37 °C. At each time point, the spread plate method was used to calculate the number of planktonic bacteria and to analyze the bacteria adhered to the surface of the samples using SEM after ultrasonic vibration [43].

The viable planktonic bacteria were calculated in the culture medium at each time point. The bacteria loosely adhered to the samples were carefully cleaned with PBS, and the adhered bacteria were separated ultrasonically for 5 min to 3 mL PBS under the frequency of 50 Hz in an ultrasonic bath (B3500S-MT, Shanghai Branson Ultrasonic Co., Shanghai, China) [44]. The solution was diluted 10 folds and plated on sheep blood Agar in triplicate, followed by incubation overnight at 37 °C. The account of CFUs was calculated according to the National Standard of China GB/T 4789.2 protocol. The samples were rinsed with PBS, fixed with glutaraldehyde solution for 4 h, then dehydrated for 10 min in gradient ethanol series, and finally dehydrated in anhydrous ethanol (twice), freeze-dried, coated with gold, and examined by SEM.

### 2.5. Cell-Surface Interactions

Human gingival fibroblasts (HGF-1) (Stem Cell Bank, Chinese Academy of Sciences, Shanghai, China) were used in the cell adhesion experiment; the cells were inoculated in 24-well plates containing samples with the concentration of 1 × 10^5^ cells/well and incubated in the Dulbecco’s modified eagle medium (DMEM; Gibco Invitrogen, Inc., Carlsbad, CA, USA) with 10% fetal bovine serum (HyClone, South Logan, UT, USA), 100 U/mL penicillin and 100 μg/mL streptomycin at 37 °C. For morphological observations, the samples were seeded with HCF-1 in 24-well plates at the concentration of 1 × 10^5^ cells per well. In different cultured periods (3, 6, 12 and 24 h), the cells were fixed for 10 min with paraformaldehyde, followed by permeabilized for 5 min by Triton X-100 (Amresco, WA, USA). Then, Rhodamine-phalloidin (Sigma, CA, USA) and DAPI were used to stain the cells for 30 min and 10 min, respectively. Finally, the cells were observed under a fluorescence microscope. The cell’s coverage area was measured by Scion image software. There were three different samples in each group, and each sample was selected randomly from five different horizons. For cell counting, the samples were carefully cleaned with PBS twice and digested using Trypsin-EDTA (TE, Gibco Invitrogen, Inc., Carlsbad, CA, USA). Cell number was counted by Cell Counting Instrument (AMQAX 1000, Thermo Fisher Scientific, Waltham, MA, USA).

The protein concentration was measured by a Bio-Rad protein analysis kit. Equivalent to polyvinylidene fluoride (PVDF) membrane (PAL). The membrane was incubated with rabbit antibody FAK, phosphorylated FAK (Tyr397) and GAPDH (Cst,1:1000 dilution) overnight at 4 °C. Then, the goat anti-rabbit antibody labeled with horseradish peroxidase was used to detect the first anti-60 min in TBST. The second binding antibody was displayed by enhanced chemiluminescence (ECL). Protein bands were quantified by TotalLabQuant of amersham in England. In the western blot experiment, after incubation for different periods (3, 6, 12, and 24 h), the protein extraction reagent was used to lyse the cells according to the protocols [45]. A Bio-Rad protein analysis kit was used to measure the protein concentration. The proteins were separated and transferred to a polyvinylidene fluoride membrane. The membranes were probed with primary antibodies overnight at 4°C, followed by incubation with horseradish peroxidase-labeled secondary antibody for 60 min and washed in TBST three times for 10 min. The proteins were measured using the Total Lab Quant (Amersham, UK).

### 2.6. Co-Culture Assay

To detect bacterial adhesion in the co-culture environment of bacteria and cells. 100 μL *S. aureus* suspension with the concentration of 1 × 10^6^ CFUs/mL and 1 × 10^4^ cells were co-cultured to each of the samples in a 24-well plate with a modified culture medium and statically incubated for different periods (3, 6, 12 and 24 h) at 37 °C. The modified culture medium included 98% regular growth medium and 2% TSB. To observe the interaction of cells and bacteria with materials in the co-culture environment, the cells and bacteria were fixed and dyed using rhodamine-phalloidin and DAPI following the above protocols, followed by stained using SYTO 9 (Invitrogen, Carlsbad, CA, USA) for 15 min and examined under a fluorescence microscope. In addition, the samples were fixed with glutaraldehyde solution for 4 h, followed by dehydrated successively with a gradient ethanol series for 10 min and dehydrated as mentioned above, and observed using SEM. To quantify bacteria adhesion for 3, 6, 12, and 24 h, the samples were carefully washed with PBS three times and sonicated and vortexed to remove adhered bacteria in 4 mL PBS. The spread plate method was used to count the adhered bacteria.

### 2.7. Statistical Analysis

The experiments were repeated in triplicate, and the data were presented as means ± standard deviations. The differences were analyzed using the one-way ANOVA and Student-Newman-Keuls post hoc tests and considered to be significant or highly significant if “*p*” values < 0.05 or 0.01, respectively. 

## 3. Results

### 3.1. Sample Characterization

**Figure 1:**
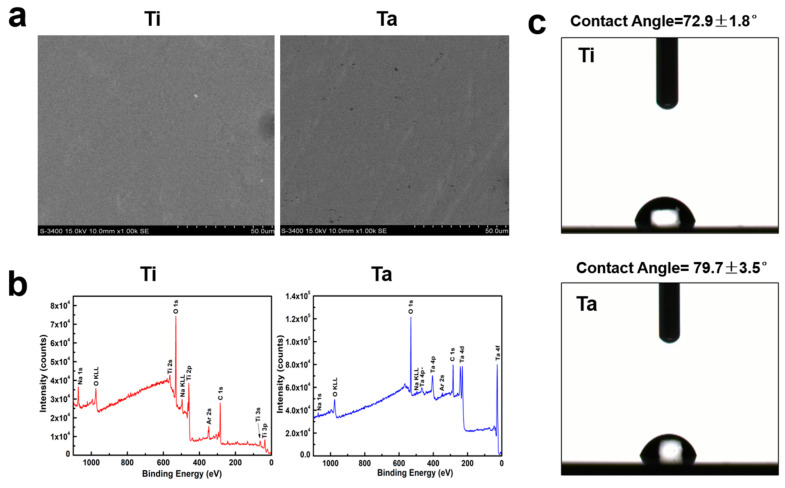
Sample characterization. (**a**) The surface topography of Ti and Ta was examined by FE-SEM. (**b**) XPS full spectra of Ti and Ta samples. (**c**) The water contact angle values for the polished Ti and Ta groups.

The surface topography of the samples is exhibited in Figure 1. SEM images of the polished Ta and Ti are illustrated in Figure 1a. The surface structure of both samples shows a flat and smooth topography. As shown in Figure 1b, characteristic peaks of Ta 4f, Ta 4d5, Ta 4s, and Ta 4p in the Ta sample were examined by XPS. The surface roughness of Ti and Ta examined by AFM was 42.95 ± 5.13 nm and 42.87 ± 5.13 nm, respectively, in our previous paper [39]. Furthermore, there was no significant difference between Ta and Ti in the surface roughness. In addition, no significant difference was observed between polished Ti and Ta, considering the surface wettability (Figure 1c).

### 3.2. In Vivo Antibacterial Property

#### 3.2.1. Radiographical Assessment

**Figure 2:**
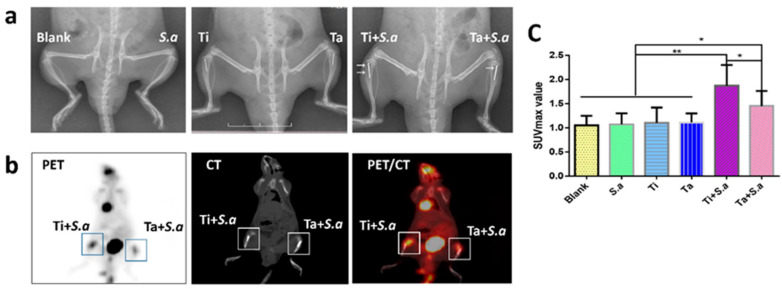
Imaging results and assessment. (**a**) X-rays of the tibia in lateral view. (**b**) PET/CT imaging of Ti + *S. a* and Ta+ *S.* groups. (**c**) SUVmax value in different groups. (* “*p*” < 0.05, ** “*p*” < 0.01).

As shown in Figure 2a, the radiographic signs of obvious osteolysis, periosteal reaction and slight soft tissue swelling were observed using X-ray after 2 weeks. There were no obvious signs of osteomyelitis in Blank, *S. a*, Ti and Ta groups. Ta + *S.*, *a* group, was demonstrated with similar radiographic signs but less than Ti + *S.*, *a* group, by X-rays. There was no deformity but swelling soft tissue in group Ta + *S. a*. In both groups, however, there were no significant advances in new bone formation, periosteal elevation and continuous deformities.

#### 3.2.2. 18F-FDG PET/CT Imaging Evaluation

PET/CT imaging showed that the uptake of 18F-FDG in Ti + *S. a* site was significantly higher than that in Ta+ *S. a* site with the mean SUVmax ratios of 1.92 (SD 0.37) and 1.43 (SD 0.34), respectively (“*p*” < 0.022) (Figure 2b,c).

#### 3.2.3. CFU of Tibia and Kirschner Wires

**Figure 3:**
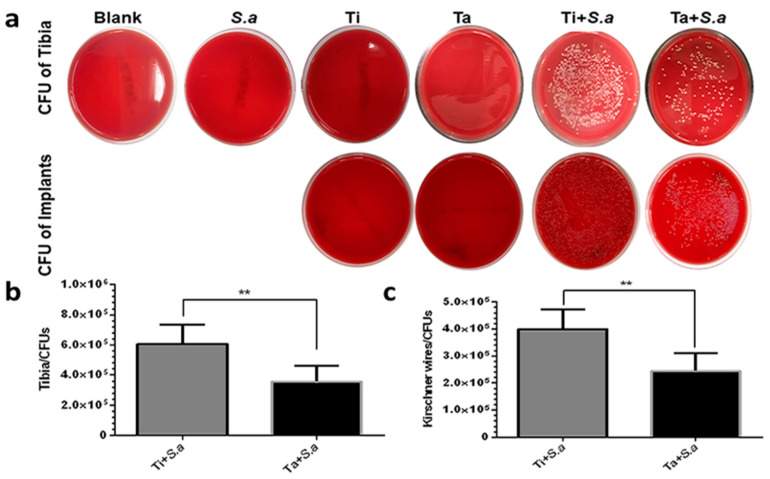
The antimicrobial results of different groups in implant-related tibia osteomyelitis model. (**a**–**c**) quantitative results of tibia powders and Kirschner wires were obtained from different groups. (** “*p*” < 0.01).

There were no bacteria cultured from the bone powder in Blank, *S. a*, Ti and Ta groups and no bacteria can be cultured from Ti and Ta groups (Figure 3a). The CFU of the tibia and Kirschner wires from group Ti+ *S. a* were more than that of group Ta+ *S. a*. (Figure 3a–c).

#### 3.2.4. Histological Evaluation

**Figure 4:**
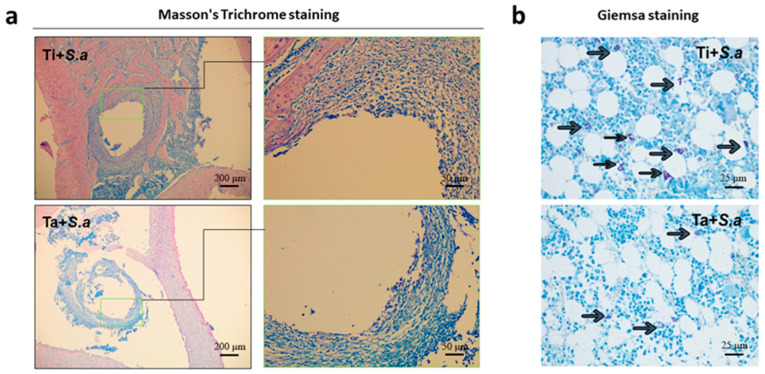
Histological evaluation of soft tissue of implant-related tibia osteomyelitis model. (**a**) Masson’s Trichrome staining of Ti + *S. a* and Ta+ *S. a* groups. (**b**) Giemsa staining of Ti + *S. a* and Ta+ *S. a* groups and scale bar = 25 μm. (the black arrow represents bacteria).

Surrounding tissues stained with Masson’s trichrome after extraction of Ti and Ta Kirschner wires (Figure 4a) showed signs of bone infection. There were plenty of fibrous tissue and inflammatory cells around without bone formation. Giemsa staining section showed that more bacteria were observed in the fibrous tissue junction and intramedullary tissue of the Ti + *S. a* group than Ta+ *S. a* group (Figure 4b). These results indicated that the severity of osteomyelitis in group Ta+ *S. a* was milder than that in group Ti + *S. a*.

### 3.3. In Vitro Anti-Biofilm Property

**Figure 5:**
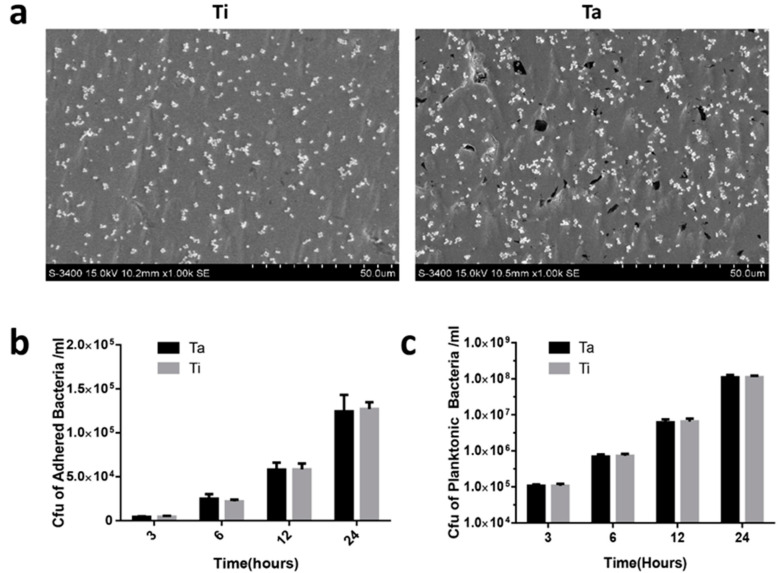
In vitro antibacterial assay. (**a**) SEM morphology of bacteria on Ta and Ti surface. (**b**,**c**) CFU of adhered and planktonic bacteria on Ta and Ti group at different time points.

SEM imaging showed that bacteria were observed uniformly adhering to the surface of Ta and Ti without obvious agglomeration (Figure 5a). There was no significant difference in bacteria adhesion of polished Ta and Ti for 3, 6, 12, and 24 h (Figure 5b). CFU was no significant reduction when comparing Ti with Ta for 3, 6, 12 and 24 h, since planktonic bacteria in both materials with plate counts at > 1 × 10^8^ CFUs/mL for 24 h (*p* = 0.969) (Figure 5c), There was no significant difference in the antimicrobial activity when comparing Ti with Ta.

### 3.4. Cell-Surface Interactions

**Figure 6:**
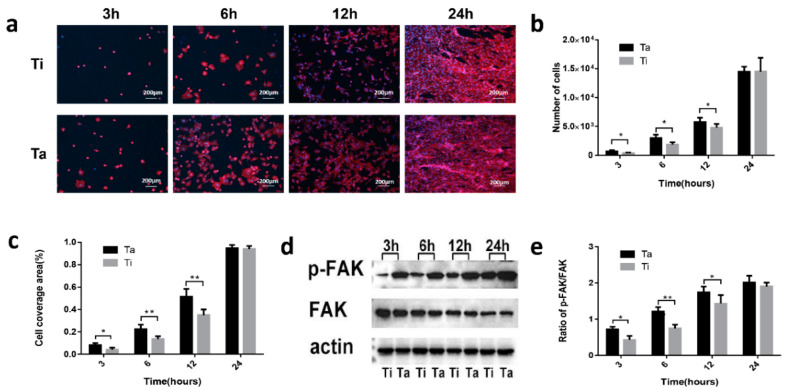
Cell-surface interactions and expressions of adhesion-related proteins. (**a**) Fluorescence images of cell-sample co-culture. Red represents actin, blue represents nuclei, and the scale bar = 200 μm. (**b**,**c**) The number of cells and cell coverage area on Ta and Ti samples at different time points. (**d**,**e**) Expressions of FAK, phospho-FAK proteins on Ta and Ti. (* “*p*” < 0.05, ** “*p*” < 0.01).

As indicated in Figure 6a, HGF-1 numbers on Ta samples were significantly more than those on Ti after 3, 6, and 12 h incubation, but no difference for 24 h. Moreover, the above statement was also demonstrated by the results of data statistics. (“*p*”< 0.05) (Figure 6b). The area spread by cells on the metal ta was larger than those on Ti after incubation for 3, 6 and 12 h and also no difference for 24 h. The cell coverage area on the surface of Ta exhibited larger than that on the Ti surface at 3, 6, and 12 h (Figure 6c), The cytoskeleton and cell morphology showed more extended and multipolar spindle shape compared with those on the Ti surface at 3, 6 and 12 h. (Figure 6a–c). In addition, Western blot confirmed that the phosphorylated FAK protein level on the Ta surface was higher than that on the Ti surface at 3, 6 and 12 h, but no difference at 24 h. (Figure 6d,e).

### 3.5. The Race between Bacterial and Mammalian Cells

**Figure 7:**
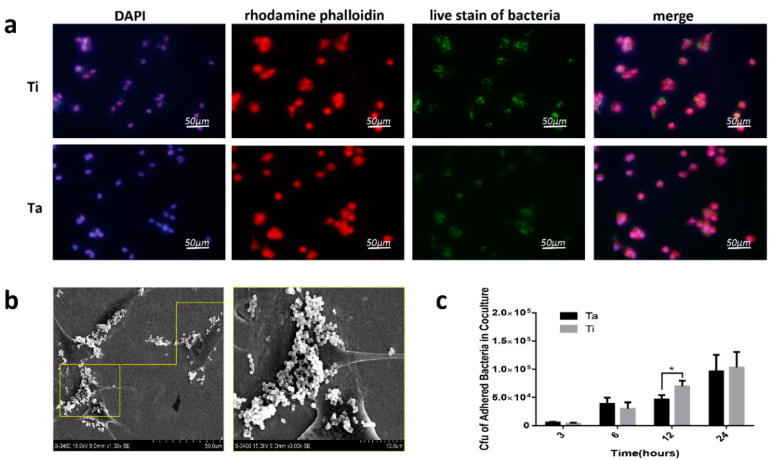
Cells and bacteria co-culture experiments. (**a**) Fluorescence images of cell-bacteria-sample co-culture. Red represents actin, blue represents nuclei, green represents live bacteria, and the scale bar = 50 μm. (**b**) Bacteria and cells on the Ta sample were examined by FE-SEM. (**c**) CFU adhered bacteria on Ta and Ti samples in co-culture. (* “*p*” < 0.05).

In observation of immunofluorescence staining shown in Figure 7a, bacteria and cells were closely attached, rather than scattered respectively, on the surface of tantalum at the early stage of co-culture (within 3–6 h), moreover, as shown in Figure 7b, the results of the SEM images further indicated that the cells earlier adhered to Ta surface, then bacteria adhered to the surface of the cell rather than Ta bare surface, bacteria agglomerated on the surface of cells rather than uniformly adhered to materials shown in Figure 7a,b. After 12 h, the cells began to necrosis. After washing, they were easy to fall off. As shown in Figure 7c, at 3 and 6 h of the co-culture system, more bacteria adhered to the tantalum surface than that titanium, but there was no statistical significance. At 12 h, more bacteria were adhering to the surface of titanium than that tantalum (“*p*” < 0.05). After 24 h, there was no significant difference in the count of bacteria.

## 4. Discussion

Titanium and its derivatives have been widely used in orthopedics and other fields because of their superior stability, and a variety of modification methods have been selected to improve the osseointegration and antibacterial properties of titanium-based implants [7,46,47,48]. However, many of the antibacterial mechanisms play a role in killing bacteria through toxicity, including reactive oxygen species (ROS) production and the destruction of DNA [49]. While these internal implant candidates are sterilized, they will inevitably hurt the normal host tissue around the implantation site, which in turn may produce negative effects such as foreign body reactions to prevent the implants from remaining in the host and failing [50]. Therefore, the preparation of an antibacterial surface must not only achieve an effective antibacterial effect but also maintain the normal functional state of tissues and cells and achieve dynamic balance. On the other hand, the selection of the modification mode, which has superior antibacterial properties and can promote the adhesion of host cells, is of great significance in improving the ability of bone integration in implants.

Tantalum has excellent corrosion resistance, and as a component of the implant, it is beneficial to reduce the local inflammatory reaction of the implant [51]. The presence of tantalum metal gives the implant good cell biocompatibility and plays an excellent role in promoting bone regeneration [52,53]. In this study, polished tantalum was selected as the experimental group, and polished titanium as the control group (Figure 1) to exclude the influence of the surface structure of materials on the results and explore the inherent biological properties of Ta. The results also proved that pure titanium could effectively promote cell adhesion, cytoskeleton extension, and diffusion (Figure 6). The authors compared the early adhesion of fibroblasts to Ta and Ti; the results showed that tantalum with good histocompatibility had better adhesion to cells (within 12 h, especially 3 to 6 h) by rhodamine-phalloidin staining and cell counting on the material surface, however, there was no difference at 24 h because of the proliferation of fibroblasts, with enough cells covering the surface of the material (Figure 6). The attachment of cells in a complex biological environment largely depends on the specific interaction between cell surface receptors and extracellular matrix (ECM) proteins. [54,55,56]. The results of Western blot for signaling proteins showed that phosphorylation of FAK was stimulated by Ta surfaces at an early stage, which is similar to the results of Zhu et al. [57], except that the object of the latter is Ta -modified micro-nanostructure titanium. Therefore, the early adhesion of fibroblasts to tantalum is not only reflected in the number of cells but also in the spreading area.

In terms of the antibacterial efficiency of tantalum, tantalum exhibited excellent antibacterial properties in the form of metal ions, and the mechanisms include DNA denaturation and destruction of the signal pathway [34,58]. It can exist in the state of tantalum oxide and nitride, and both of them have been found to have certain antibacterial activity [59,60]. In addition, Zhang et al. observed that *F. nucleatum* (*F. n.*) and *Porphyromonas gingivalis* (*P. g.*) also adhere less to the tantalum coating [61]. However, Harrison et al. observed that Ta was not demonstrated to possess inherent antibacterial activity compared with Ti [37]. In vivo studies and clinical studies reported a decline in infection rates in cases of revision hip arthroplasty with porous tantalum [62,63]. while in the rabbit prosthesis model, porous tantalum cannot prevent nail infection [64]. In this study, at the early experimental stage, the differences in antimicrobial properties between Ta and Ti were found in a rat implant-related infection model (Figure 2). It is worth mentioning that the X-ray findings in vivo are not very obvious because this study mainly focuses on the early manifestations of osteomyelitis models, mainly local low-density images in the bone marrow cavity. There was no obvious periosteal reaction, bone capsule formation, and dead bone formation, and it was to evaluate the severity of early osteomyelitis more objectively. Therefore, PET/CT was used to evaluate the severity of osteomyelitis in the Ta-Kirschner wires group, which showed that the uptake of 18F-FDG was lower in Ta- the Kirschner wires group. The results of histological examination and microbiological cultures also showed that the number of bone destruction, inflammatory cell infiltration, and bacteria in the Ta group were less than those in the Ti group (Figure 3 and Figure 4). To further explore the potential mechanisms behind the inconsistent infection degree of tantalum and titanium in vivo, the authors designed and carried out in vitro experiments. In the subsequent experiments, it was observed that pure tantalum had no significant inhibitory effect on *S. aureus* in vitro compared with titanium, indicating that tantalum does not exhibit intrinsic antimicrobial properties (Figure 5). Conditional pathogens on implant surfaces, operating rooms, surgical equipment, surgeons, patients themselves, contaminated disinfectants, and others can reach the implant surface through direct contact, hematogenous dissemination, or endogenous displacement. The characteristics of implant materials and implant site are important factors affecting the occurrence of infection [65]. The study has confirmed that bacterial colonization and adhesion are the initial factors of orthopedic implant infection. After implantation, proteins (such as fibrinogen, bole in, compliment, fibronectin, etc.) cells (such as fibroblasts, neutrophils, mesenchymal stem cells, etc.) from blood or tissue fluid quickly adhere to the implant surface to form a temporary surface matrix, at the same time, Local coagulation reactions and complement systems are activated, then immune cells are activated [66]. Therefore, in this study, the authors established an in vitro model of co-culture with bacteria and cells to simulate the microenvironment in vivo to some extent (Figure 7). Time points including 3, 6, 12, and 24 h were selected to simulate the early adhesion in vivo and the characteristics of the interaction discussed between bacteria and cells on the tantalum surface. To simulate peri-operative infection, in the co-culture experiment, bacteria were permitted to adhere for 2 h before cell adhesion. In co-culture experiments, cells are planned to be stained for different periods (3, 6, 12, and 24 h). However, it was found that after the co-culture of bacteria and cells for more than 6 h, cells began to damage or even die. Cells will be lost in the process of washing and cannot be evaluated objectively. Surprisingly, early-stage live bacteria staining demonstrated that the bacteria were extremely close to the cells. Moreover, SEM observation more intuitively revealed that bacteria were more likely to adhere to the cell surface and reunite in the co-cultured instead of the surface of Ta (Figure 7a,b). The study has confirmed that bacterial colonization and adhesion are the initial factors of orthopedic implant infection. Various factors, such as polarity, van der Waals force, and hydrophobicity, together regulate the initial bacterial adhesion [67]. This means that the early adhesion of bacteria or cells to the implant surface has a great impact on the occurrence of implant infection. More bacteria adhered to the Ta because more cells existed within hours. As the experiment continued, at 12 h, the cells began to exfoliate, and cell exfoliation also took away the bacteria attached to them. The bacteria on the surface of cells were taken away after washing. Considering that there is a powerful immune system in the normal body environment, including a variety of immune cells that will destruct bacteria [68], which cannot be reflected in this co-culture model, therefore, at 24 h, the cells were almost exhausted and could not be compared, and bacteria have completely taken over the position.

Previously, the theory of “the race for the surface” has been put forward and embraced by some researchers [69,70,71,72]; this theory properly explains the phenomenon of competitive colonization of bacteria and cells on the implant surface. Moreover, the mainstream view was that host cell attachment helps to prevent infection (reduce the incidence of infection) [69,71,73]. On the basis of this theory, foreign bodies could trigger the competition between host cells and bacteria to colonize the implant surface. [40,41,42]. In other words, when the host cells win the competition, the cells will occupy the surface, thus inhibiting the adhesion and colonization of bacteria. On the contrary, bacteria will invade the surface of the implant to form biofilms, and the host cells will be suppressed by virulent bacterial substances, which eventually lead to infection. The results of this competition greatly determine the infection severity and inhibit organizational integration. Phenomena that bacteria adhere to the cell surface preferentially rather than directly on the material surface were observed in these experiments but have not been paid special attention to. The authors hold the opinions that these phenomena are extremely surprising and interesting and have remarkable significance. In the early stage, tantalum has the superior capability to promote cell adhesion, making cells preferentially adhere to the surface of Ta, and then bacteria are more likely to adhere to the cells that have already adhered to the surface of Ta rather than the exposed surface of Ta. Over time, the cells die and fall off, causing the bacteria attached to their surfaces to fall off as well. As a result, the chance of bacteria directly adhering to Ta is greatly reduced, which is helpful in reducing the production of biofilm and the occurrence of implant infection. In addition, bacteria adhered to the cell surface are more likely to be involved by antibiotics and immune cells regardless of whether they fall off or not [74]. Considering the different antibacterial properties of tantalum in vivo and in vitro, some scholars have also given relevant explanations for the phenomenon that tantalum has a certain capability to resist infection in the body. Schildhauer et al. reported that porous tantalum effectively activates the immune system represented by leukocytes, creates a microenvironment conducive to killing bacteria, and may enhance the defense ability of local hosts [75]. In addition, Yang et al. observed that the Ta nanomembrane can significantly improve the ability of neutrophils to phagocytize bacteria and promote macrophages to release pro-inflammatory cytokines. Thus, the host can exterminate bacteria by regulating the immune environment [76]. Combined with in vitro experimental results, the bacteria peeled off with the cells exfoliation will be attacked by many immune cells activated in vivo microenvironment, and tantalum possesses dual effects on eliminating bacteria in addition to its immune initiating ability, to achieve the role of preventing implant infection.

This study has some limitations, this study found the above surprising phenomenon through in vitro immunostaining and SEM observation, but more in vitro, ex vivo, and in vivo experiments are needed to further explore this phenomenon. Moreover, the authors chose the most common pathogen of osteomyelitis (*S. aureus*) as the research object. In future work, the authors will further study the interaction between Gram-negative bacteria and cells on the surface of Ta. In addition, the authors will continue to explore and improve the mechanism behind this phenomenon in the following work. This article is more inclined to put forward the concept, appealing for more attention to focus on the appearance.

## 5. Conclusions

The original purpose of the fabrication and development of antibacterial implant materials is to seek materials with both excellent antibacterial properties and host tissue protection. The results of this study show that tantalum has good biological compatibility and contributes to cell adhesion and proliferation. In addition, although tantalum does not have the inherent antibacterial ability, the discovery that bacteria tend to adhere to the cell surface rather than the tantalum surface further explains the reason for the difference between tantalum antibacterial results in vivo and in vitro, which provides a certain basis for the fabrication of tantalum-based antibacterial implants in the future. In addition, this finding also brings a new perspective to the manufacture of orthopedic antibacterial implant materials. On the one hand, more focus needs to be paid to the interaction between the host, bacteria, and implants, instead of directly targeting bacteria, on the other hand, biomaterials with good cell adhesion ability deserve more attention, because they may exert their ability to resist bacterial infection through the way found in this study.

## Figures and Tables

**Table 1:** Details of animal experiments.

Group	Number (n)	Left/Right	Implant	Inoculation	Abbreviation
I	10	right leg	no	10 μL PBS	Blank
		left leg	no	*S. a* 10^3^ CFU/10 μL	*S. a*
II	10	right leg	Ti wire	10 μL PBS	Ti
		left leg	Ta wire	10 μL PBS	Ta
III	15	right leg	Ti wire	*S. a* 10^3^ CFU/10 μL	Ti + *S. a*
		left leg	Ta wire	*S. a* 10^3^ CFU/10 μL	Ta + *S. a*

## Data Availability

Not applicable.
